# Left thoracoscopic approach in the supine position for torsion of the residual esophagus after esophagectomy: a case report

**DOI:** 10.1186/s40792-022-01430-9

**Published:** 2022-05-04

**Authors:** Kazuya Yamaguchi, Shigeo Haruki, Masayoshi Sakano, Kunihito Suzuki, Akinori Miura

**Affiliations:** 1 Department of Esophageal Surgery, Tokyo Metropolitan Cancer and Infectious Diseases Center, Komagome Hospital, 3-18-22 Honkomagome, Bunkyo-ku, Tokyo, 113-8677 Japan; 2 Department of Gastrointestinal Surgery, Tokyo Medical and Dental University, 1-5-45 Yushima, Bunkyo-ku, Tokyo, 113-8510 Japan

**Keywords:** Left thoracoscopic, Anastomosis, Stenosis, Torsion, Esophagectomy

## Abstract

**Background:**

Anastomotic stenosis can occur after esophagectomy and gastric tube reconstruction. The effective surgical treatment for refractory anastomotic stenosis, which seems difficult to resolve with endoscopic treatment, has not been established. We report a case of refractory stenosis due to esophageal torsion in which reconstructive surgery was possible using a left thoracoscopic approach in the supine position.

**Case presentation:**

A 72-year-old man who underwent thoracoscopic subtotal esophagectomy and retrosternal gastric tube reconstruction for esophageal cancer 6 months previously presented to us. Postoperative endoscopy revealed that the residual esophagus was twisted approximately 360°, just above the anastomotic site. Conservative endoscopic treatment failed to improve the condition due to severe passage obstruction, and reconstructive surgery was repeated. Surgery was performed in the supine position using a left thoracoscopic approach. The entire circumferences of the gastric tube and residual esophagus were dissected from the inferior mediastinum to the top of the sternum, with focus on preserving the right gastroepiploic vein and gastric-tube wall. Subsequently, laparoscopic surgery was performed to remove the gastric tube from the thoracoabdominal junction. After separating the esophagus on the oral side of the torsion from the left cervical wound, the abdomen was opened, the gastric tube was pulled out through the abdominal wound, and adhesions in the abdominal cavity were peeled off to raise the gastric tube cranially via the retrosternal route. An end-to-side anastomosis was performed using a circular stapler, and the esophageal torsion and previous anastomosis were resected. Oral intake was resumed on the 7th postoperative day, and the patient was discharged on the 38th day.

**Conclusions:**

After subtotal esophagectomy and retrosternal gastric tube reconstruction, the left thoracoscopic approach is one of the most minimally invasive approaches and is especially useful for preserving the right gastroepiploic artery and veins and for mobilizing the gastric tube wall.

## Background

Complexities around the anastomosis after esophagectomy is a major cause of oral intake difficulty. Most cases involve scar stenosis due to anastomotic leakage or impaired blood flow; however, torsion of the esophagus or gastric tube, with a twisted anastomosis, causes passage obstruction.

Endoscopic dilation using a balloon is often performed for benign anastomotic stenosis after esophagectomy and gastric tube reconstruction [1–3]. Although endoscopic treatment is adequate for resolving stenosis due to torsion of the residual esophagus or gastric tube, passage obstruction due to twisting is often intractable. No effective surgical intervention has been established for refractory stenosis, which is difficult to resolve using endoscopic treatment.

We report a case of refractory stenosis due to residual esophageal torsion, which was minimally invasively reoperated using a left thoracoscopic approach in the supine position.

## Case presentation

The patient was a 72-year-old man. Upper gastrointestinal endoscopy for alcoholism revealed thoracic esophageal cancer. His medical history included hypertension, nephrectomy for right kidney cancer, mild dementia, cholangitis, alcoholism, and lower limb movement disorder. We diagnosed the patient with esophageal cancer Mt, cT1b, N1, M0, cStage I (Union for International Cancer Control tumor-node-metastasis classification, 8th edition). Thoracoscopic subtotal esophagectomy, three-field lymph node dissection, and retrosternal gastric tube reconstruction were performed. Esophagogastric end-to-side anastomosis was performed on the left side of the neck using a circular stapler. The insertion hole of the circular stapler was closed using a linear stapler. After the anastomosis, the gastric tube was pulled out from the abdomen to straighten the residual esophagus.

Regular endoscopy performed on the day after surgery revealed that the residual esophagus was twisted 360° and was anastomosed. Since it was possible to pass a 5.8-mm–diameter scope, we followed up the patient without reoperation. Oral intake was initiated on postoperative day (POD) 8, and the patient could gradually be fed orally. The patient was discharged on POD 17, with the possibility of enteral nutrition.

**Fig. 1:**
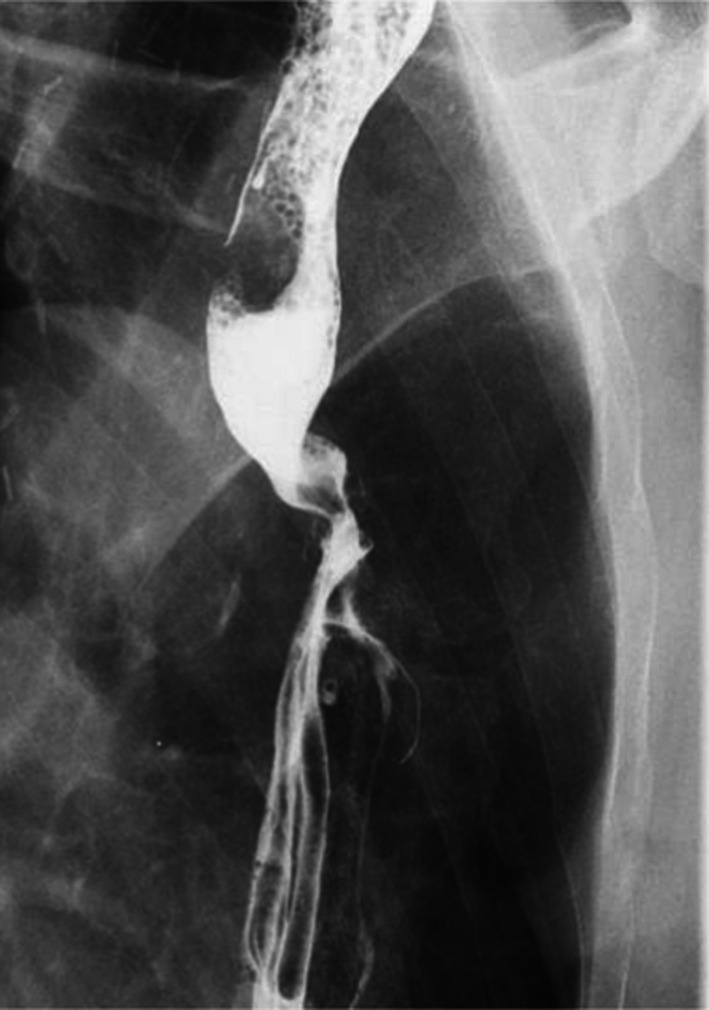
Esophagography after the initial operation. Right anterior oblique view. The residual esophagus is twisted at the height of the aortic arch, blocking the passage of barium

**Fig. 2:**
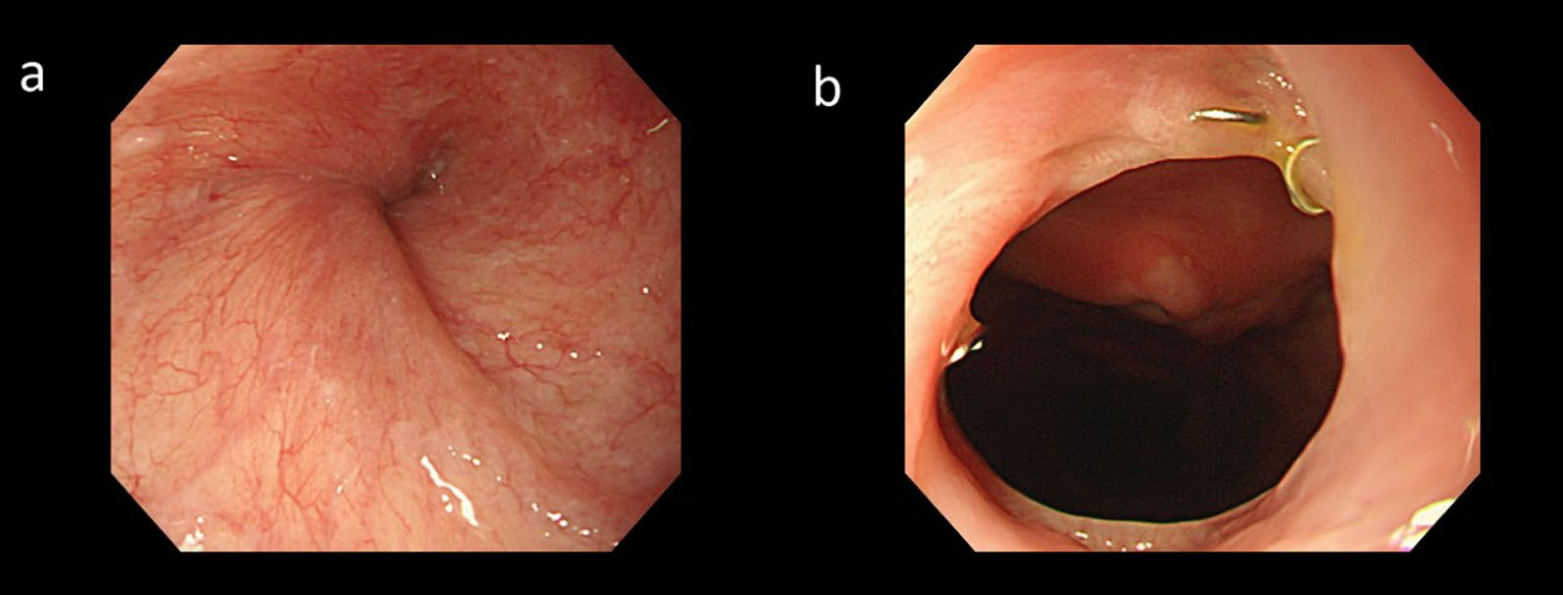
Endoscopy after the initial operation. **a** Esophageal torsion. **b** Esophagogastric anastomosis. Just above the anastomotic site, the remaining esophagus is twisted 360° and stenosed. An 8.9-mm endoscope could pass through the esophagus without resistance

**Fig. 3:**
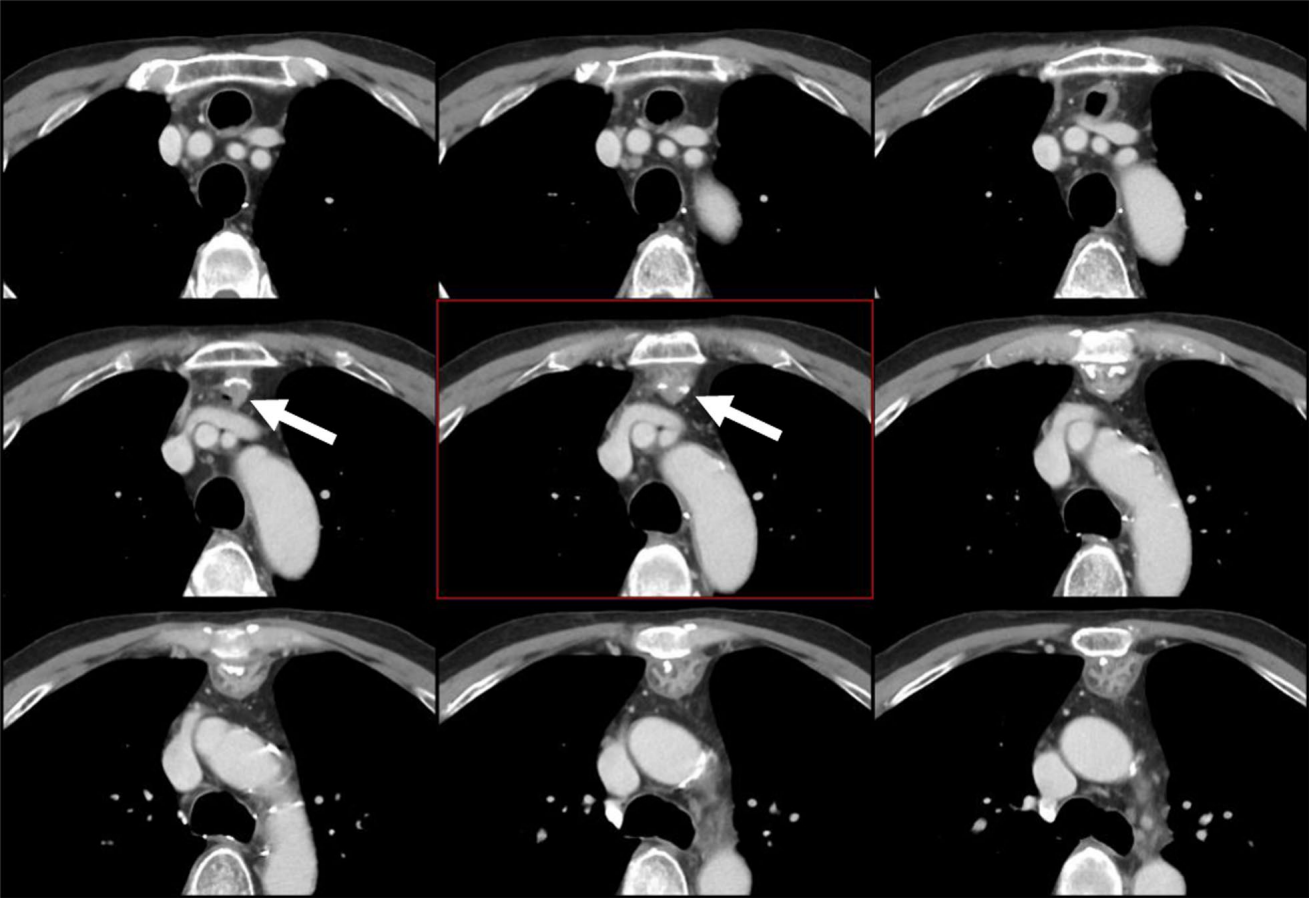
Computed tomography showing that the anastomotic ring (white arrow) is 4 cm caudal to the top of the sternum and that the oral side of the esophagus is dilated

However, oral nutrition gradually became impossible, and the patient was readmitted 3 months postoperatively. Esophagography revealed a spirally twisted esophagus that allowed only a small amount of barium to pass through (Fig. 1). Although endoscopy clearly confirmed the presence of torsion just above the anastomosis (Fig. 2), a scope with 8.9 mm diameter could pass through the esophagus without resistance. Endoscopic dilation to 15 mm was performed using a balloon; however, the esophagus was only slightly opened, and the stenosis recurred. Computed tomography showed that the anastomotic ring was 4 cm caudal to the top of the sternum and the residual esophagus was dilated (Fig. 3). All nutrition was provided enterally, and outpatient rehabilitation was continued. We expected that swallowing rehabilitation would improve oral intake.

**Fig. 4:**
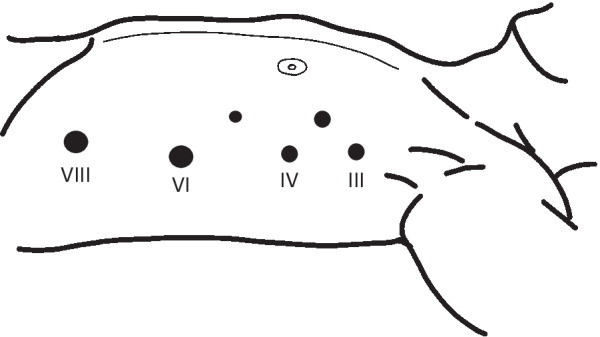
Port placement. Ports are placed in the anterior axillary line between the 3rd, 4th, 6th, and 8th intercostal muscles, with 5-mm and 3-mm ports added to the precordium side during surgery

**Fig. 5:**
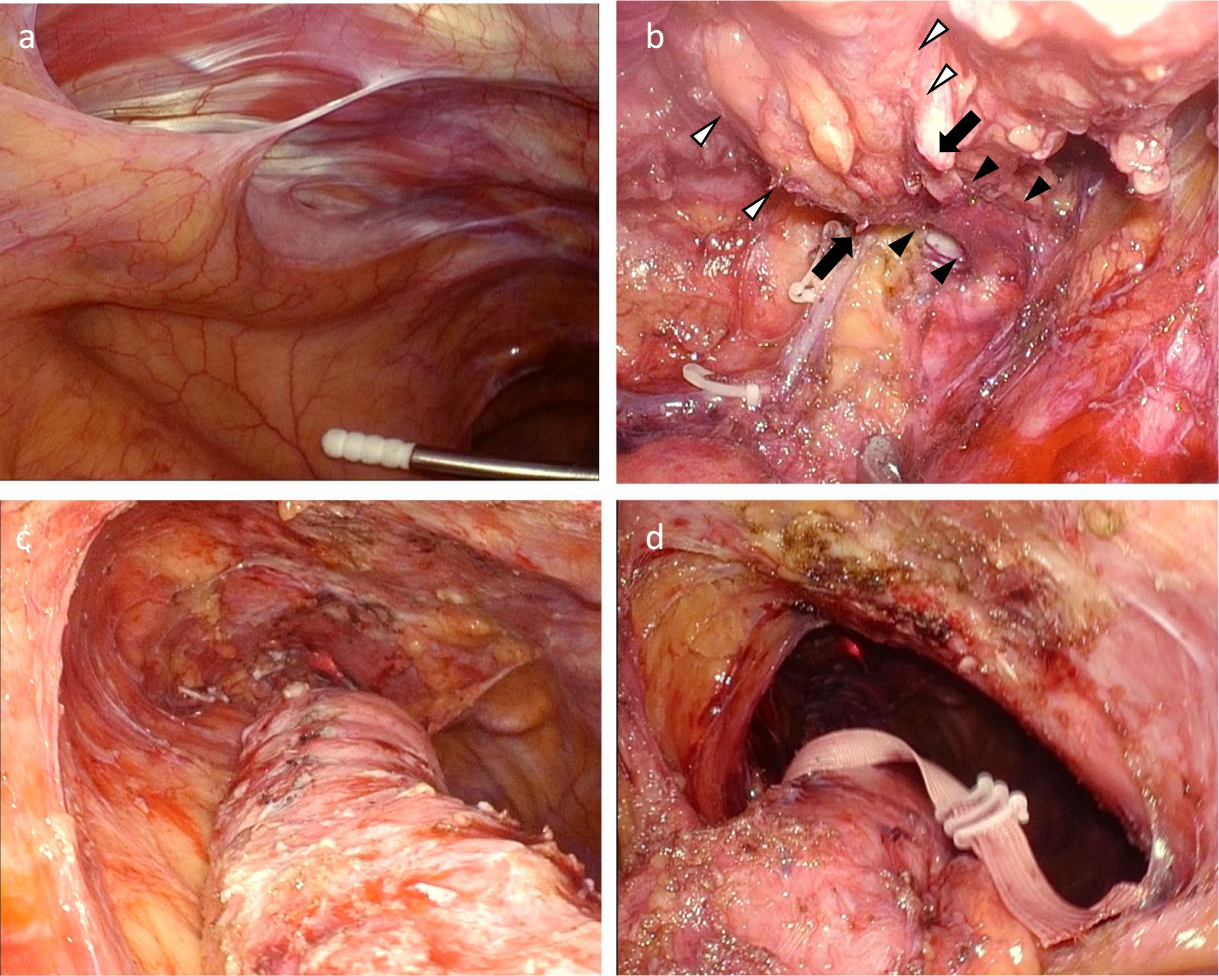
Intraoperative photo. **a** The gastric tube is confirmed between the pericardium and chest wall. **b**: After peeling around the anastomotic site. The black arrowhead is the esophagus. The white arrowhead is the gastric tube. The black arrow is the anastomotic part. **c** After exfoliation around the gastric tube in the thoracic cavity. The right pleura is preserved. **d** Observation from the abdominal cavity. It was confirmed continuously from the entrance of the retrosternal route to the neck

However, the patient encountered difficulty in eating for 6 months postoperatively, and we resorted to surgical treatment. We planned to remove the torsion and re-anastomose the esophagus and gastric tube. The left thoracoscopic approach was used in the supine position. Thoracoscopy was performed using an artificial pneumothorax of 8 mmHg and right one-lung ventilation. Ports were placed in the anterior axillary line between the 3rd, 4th, 6th, and 8th intercostal muscles, with 5-mm and 3-mm ports added to the precordium side during surgery (Fig. 4). When the left thoracic cavity was confirmed, the left edge of the gastric tube was confirmed behind the sternum (Fig. 5a). Attention was focused on preserving the right gastroepiploic vessels and gastric tube wall, and an incision was made in the pleura on the dorsal side of the gastric tube to separate it from the pericardium. Subsequently, an incision was made in the ventral pleura of the gastric tube, which was separated from the sternum. During the initial operation, the staples on the lesser curvature side were buried; however, they were found to have adhered to the sternum and were carefully peeled off. A high degree of adhesion was observed around the anastomotic site. Torsion of the esophagus was not clearly identified from within the thoracic cavity due to scarring around the anastomotic site (Fig. 5b). Finally, the entire circumferences of the gastric tube and residual esophagus were released from the inferior mediastinum to the upper end of the sternum, and the right pleura was preserved (Fig. 5c).

**Fig. 6:**
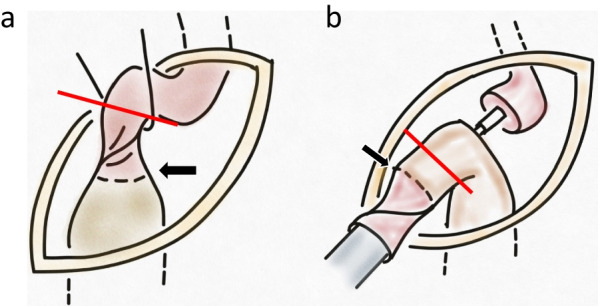
Left neck operation. The upper side of the figure is the cranial side, and the lower side is the caudal side. **a** The esophagus and gastric tube are pulled from the neck. Black arrow is the old esophagogastric anastomosis. Red line is dissection on the oral side of the esophageal twist. **b** A circular stapler is inserted from the esophageal stump, The twisted part of the esophagus was easily dilated and the body of the circular stapler could pass through. An esophagogastric end-to-side anastomosis was performed on the caudal side of the old anastomosis. The insertion hole of the circular stapler was closed using a linear stapler (red line)

Subsequently, laparoscopic surgery was performed, and the area around the gastric tube at the thoracoabdominal transition peeled off and continued with the layer from the thoracic cavity (Fig. 5d). Simultaneously, left cervical surgery was performed to remove the residual esophagus, which was linked to the thoracic cavity. The esophagus was cut off on the oral side of the torsion (Fig. 6a). The gastric tube was raised to the left neck again through the retrosternal route, and a circular stapler was inserted from the cut part. The twisted part of the esophagus was easily dilated and the body of the circular stapler could pass through. An esophagogastric end-to-side anastomosis was performed on the caudal side of the old anastomosis. The old anastomosis and esophageal torsion were removed, and the insertion hole of the circular stapler was closed using a linear stapler (Fig. 6b). Finally, the gastric tube was pulled down from the abdominal wound to straighten the residual esophagus, and no torsion was confirmed. The operative time was 6 h 49 min, and the bleeding volume was 240 mL.

**Fig. 7:**
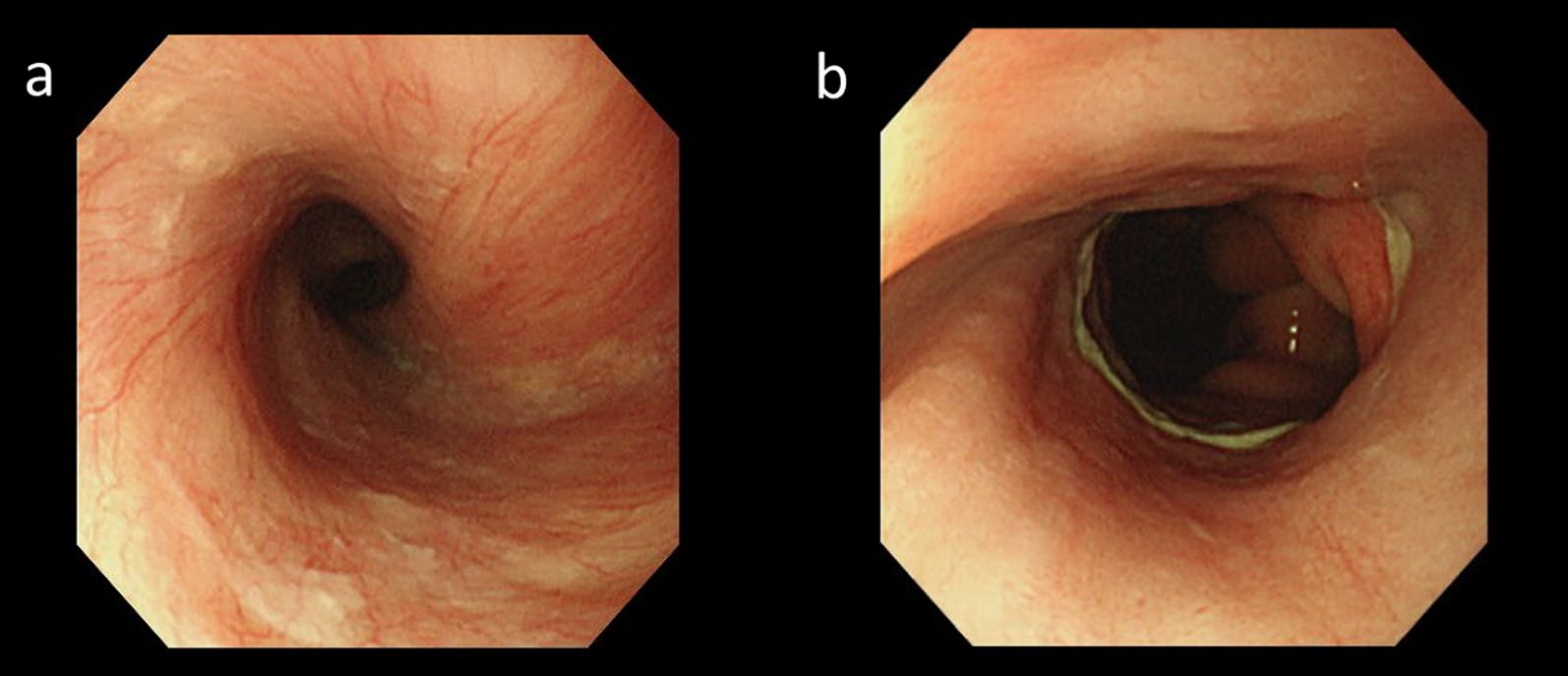
Postoperative endoscopy. **a** Residual esophagus. **b** Re-anastomosis. The stenosis is improved

Postoperative endoscopy revealed relief of the torsion-induced stenosis (Fig. 7). Oral intake was resumed on POD 7. Although postoperative aspiration pneumonia developed, the patient was discharged on POD 38.

## Discussion

Endoscopic balloon dilatation or bougie intubation is generally performed first for benign anastomotic stenosis after esophagectomy and gastric tube reconstruction. Local steroid injections of as well as radial incision and cutting are often performed for refractory stenosis [1]. Temporary stent placement for refractory benign stenosis has also been reported [4]. Although there are reports of percutaneous plasty for esophagocolonic anastomosis in patients who were refractory to endoscopic treatment, no surgical method has been established [3].

In this case, passage was blocked by esophageal torsion just above the anastomotic site. Torsion of the gastric tube and esophagus occasionally occurs after esophagectomy. There are several reports on the prevention of gastric tube torsion during gastric tube reconstruction [5, 6]. We also pulled out the gastric tube through the retrosternal route while checking the staple line on the gastric lesser curvature using the operator's hand. Postoperative endoscopy showed that the gastric tube was not twisted. Thus, the residual esophagus was already twisted 360° before the anastomosis, and the anastomosis may have been performed without confirming whether there was no twisting. Before performing anastomosis, it is important to establish that the remaining esophagus is not twisted. If the residual esophagus is too long, it can get twisted when the gastric tube is pulled after anastomosis; therefore, the residual esophagus must be of appropriate length.

Even if twisting exists, it may allow some degree of passage, and in many cases oral intake is possible. In the present case, there was no resistance to the passage of the endoscope, and it was expected that by continuing swallowing rehabilitation and the recovery of vocal cord paralysis there would be reduction in the stenosis. However, the performance status of the patient was originally low, and no improvement was seen after 6 months, leading to the decision to perform a reoperation. Because the anastomotic site was in the mediastinum, not in the left neck, reconstruction surgery including mediastinal surgery was considered necessary.

Reoperation after gastric tube reconstruction and esophagectomy is highly invasive and carries a risk of fatal complications. When total gastrectomy was performed, the reported complication rate was 83%, including complications such as long-term air leaks, vocal cord paralysis, cervical anastomotic leakage, respiratory failure, and dehiscence of open wounds. This procedure is considered highly invasive because of the severe adhesion around the gastric tube and the high risk of extensive intraoperative bleeding [7].

Further, median sternotomy is often performed as an approach to gastric resection for retrosternal gastric tube reconstruction, with full or partial resection of the gastric tube [8]. Postoperative pain often negatively affects respiration when median sternotomy is performed [9].

Therefore, various methods have been attempted as minimally invasive approaches for treating gastric tube cancer after esophagectomy and for retrosternal gastric tube reconstruction.

Kato et al. reported that the gastric tube could be resected minimally invasively using hand-assisted right thoracoscopic surgery. However, he highlighted that adhesions in the right thoracic cavity could complicate the procedure [10].

A new operative technique for gastric tube cancer involves lifting the anterior chest wall and videoscope-assisted surgery [11, 12]. However, it has been highlighted that the procedure is difficult due to the presence of marked adhesion around the gastric tube [12].

Recently, Horie et al. reported a method for removing the gastric tube in the supine position using a left thoracic approach. They reported the method as safe, with the provision of a favorable surgical view [13]. In all the above surgeries, it was assumed that the gastric tube was removed.

After excising the torsion, we performed re-anastomosis using the gastric tube. Therefore, it is necessary to maintain the right gastroepiploic artery and vein on the left side of the gastric tube and prevent damage to the gastric tube wall as much as possible. The left thoracoscopic approach in which the right gastroepiploic artery and vein are in front of the surgical field and the left pleura is resected together to ensure vessel preservation was performed. By exposing the ventral sternum and dorsal pericardium, we could secure the gastric tube without exposing the right gastroepiploic artery and veins. Additionally, in cases where it was difficult to move the forceps into the anterior mediastinum, the anterior mediastinum could be approached by inserting a new port on the ventral side.

In the present case, the gastric tube was long enough for re-anastomosis; hence, esophagogastric end-to-side anastomosis could be repeated. If the gastric tube length was insufficient, we would have considered hand-sewn end-to-end anastomosis or the altering of the reconstruction route in front of the chest wall, with the insertion of the free jejunum.

Furthermore, in this case, the initial surgery was accompanied by right vocal cord paralysis; therefore, it was necessary to consider the risk of complications involving left vocal cord paralysis during the reoperation. The area around the residual esophagus was carefully removed during the operation, and left vocal cord paralysis was not observed after surgery.

Although this patient developed postoperative aspiration pneumonia, which might have been due to a decrease in performance status owing to lower limb movement disorder, no other major complications were observed. In the supine position, the left thoracoscopic approach is believed to be minimally invasive and effective for preserving the gastric tube and vessels after esophagectomy with retrosternal gastric tube reconstruction.

## Conclusion

The left thoracoscopic approach is one of the most minimally invasive approaches, with a favorable surgical view after retrosternal gastric tube reconstruction. This approach is also useful for preserving the right gastroepiploic artery and veins as well as for safely removing the gastric tube for re-anastomosis.

## Data Availability

Datasets supporting the conclusions of this study are included within the article and its additional files. Datasets are available from the corresponding author, Kazuya Yamaguchi, upon reasonable request.

## Abbreviations

POD: Postoperative day
CT: Computed tomography
